# Understanding flow dynamics, viability and metastatic potency of cervical cancer (HeLa) cells through constricted microchannel

**DOI:** 10.1038/s41598-018-35646-3

**Published:** 2018-11-26

**Authors:** Binita Nath, Asif Raza, Vishal Sethi, Amaresh Dalal, Siddhartha Sankar Ghosh, Gautam Biswas

**Affiliations:** 1 Department of Mechanical Engineering, Indian Institute of Technology, Guwahati, 781039 India; 2 Department of Bioscience and Bioengineering, Indian Institute of Technology, Guwahati, 781039 India

## Abstract

To understand the burgeoning challenges of metastasis, a microchannel of 35 μm diameter, constricted to 7 μm for a distance of 200 μm in a total length of 3 mm, was designed and fabricated using a mask aligner made of polydimethylsiloxane (PDMS) to mimic *in vivo* capillaries. A thin glass cover-slide was mounted on top to monitor the motion of single or aggregated malignant HeLa cells (size 17–30 μm) microscopically through the constricted microchannel at a constant flow rate of 30 μl/h. Quantitative deconvolution of high-speed videographs of a single cell of 30 μm revealed cellular deformation while passing through constriction, having elongation index, average transit velocity and entry time of 2.67, 18 mm/s and 5.1 ms, respectively. Morphological analysis of live and apoptotic cells by dual staining with Acridine Orange/Ethidium Bromide demonstrated retention of a significant viable cell population after exit through the constriction and a viability index of 50% was quantified by dye exclusion assay. The cumulative data for microfluidic parameters, morphology and relevant metastatic MMP2 gene expression efficiency measured by real-time polymerase chain reaction revealed retention of virulence potency that could possibly cause metastasis, would be beneficial in developing futuristic MEMS device for cancer theranostics.

## Introduction

Even in this modern era of cancer therapeutics, scientists and oncologists have not been able to resolve the mystery of metastatic cancer, which causes high mortality worldwide. In many instances, cancer is found to be reinvigorated in the other parts of the body, where chemotherapeutic drugs cease to work. Escalation of doses is often seen to damage healthy cells and worsen the prognosis^1^. A population of small, loosely bound clusters of cancer cells deriving from the primary tumour sites, also known as circulating tumour cells (CTCs), are able to stray away from the aggregate cluster through motion in the bloodstream or the lymphatic system, causing metastasis^2^. Hence the analysis of CTC dynamics plays a paramount role in understanding the nature of parent tumour aggregates^3^. CTCs are often utilized as effective blood-borne biomarkers to enhance treatment methodologies^4^ and curtail metastasis^5^. They also provide a measure of cancer genotype during therapy and phases of disease progression. About 5–50 CTCs per 5 ml of blood in the bloodstream of several cancer patients^2^ have been reported to pass through even micron-sized capillaries and undergo great deformation, with a pronounced impact on their morphology. The isolation of CTC clusters from the peripheral blood of cancer patients has established their presence in the blood flow and their ability to pass successfully through the capillary beds and finger capillaries^6–13^. A thorough investigation of these aspects may lead to a better estimation of the nature of drugs and requirements of modalities to manage the treatment.

In the past few decades, several efforts have been made to elucidate the role of CTCs in seeding metastasis, where two or more CTCs form clusters, and these clusters are reported to be strong initiators of metastasis compared with singlets^6,14–16^. The flow of cells in a capillary is complex owing to the size of the capillary (5–10 μm), and if cancer cells were to exhibit increased deformability they would have a higher probability of migrating to other parts of the body^17^. However, the nucleus is approximately 5–10 times stiffer than the surrounding cytoskeleton and thereby resists large changes in shape^18^. Therefore, the nucleus is thought to be the rate-limiting organelle regarding migration through small openings. Yamauchi *et al*. studied the cytoplasmic and nuclear deformation of cancer cells migrating in capillaries by injecting cells into the hearts of nude mice^19^. It is often challenging to study the behaviour of CTCs in real conditions, hence constricted microfluidic channels with a width smaller than the diameter of the cells are often fabricated using standard microfabrication techniques, as they are efficient in providing an environment to mimic *in vivo* capillaries^20^. Such constricted channels have been used to evaluate the mechanical properties of red blood cells (RBCs)^21–25^, leukocytes^26–28^ and cancer cells^29–31^. For example, Hou *et al*.^30^ demonstrated experimentally a simple microfluidic channel to distinguish the difference in stiffness between benign and breast cancer cells. Several other groups have studied the behaviour of CTCs in capillaries computationally^31,32^. Numerical adaptation to study the dynamics of CTCs allows precise control over the various important hydrodynamic parameters to elucidate the transit behaviour of the CTC clusters only. The recent experimental and numerical work of Au *et al*.^31^ demonstrated the flow of CTC aggregates through capillaries and negated restriction of the passage of CTCs through capillaries owing to the difference between the size of the tumour cells and the diameters of the capillaries^33^.

**Figure 1:**
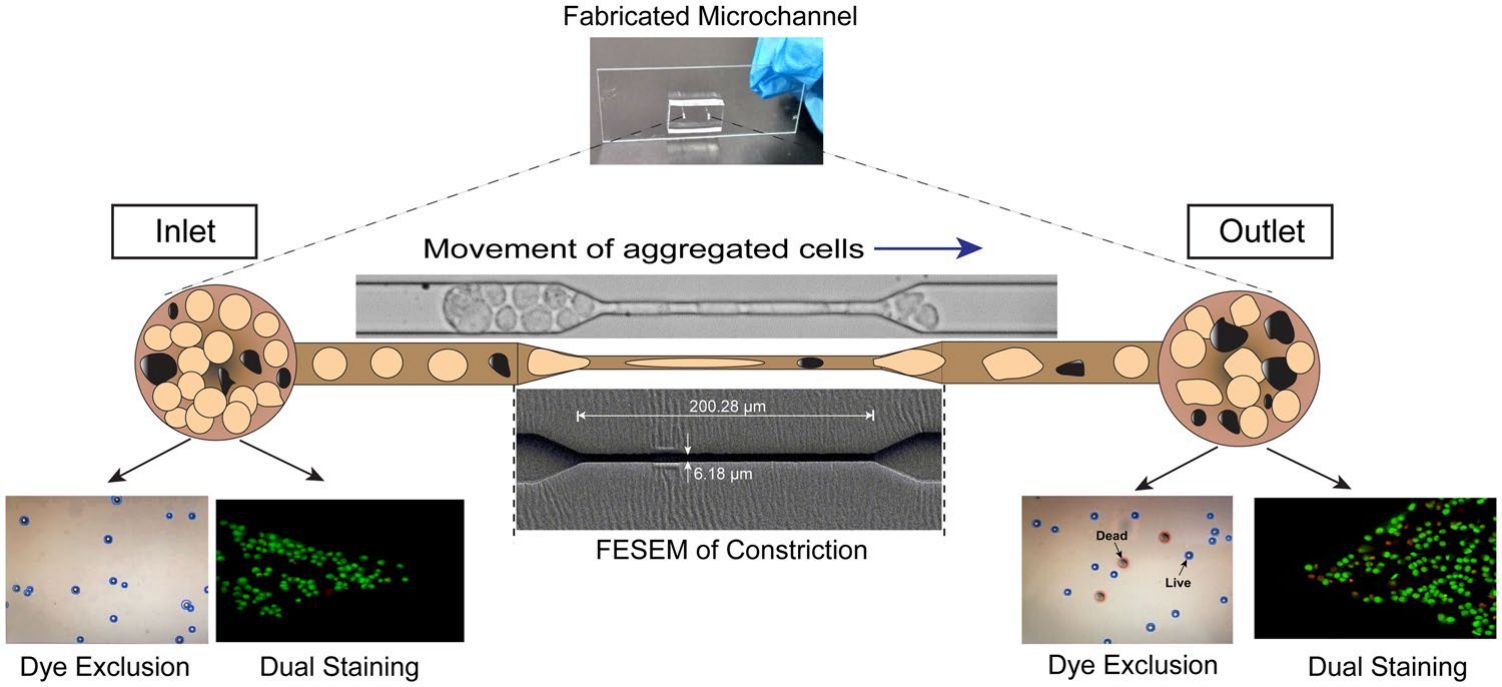
Schematic representation of the motion of aggregated HeLa cells passing through a microcapillary to seed distant metastasis.

The main objective of this work was to elucidate the flow behaviour of metastatic cancer cells experimentally, similar to CTCs, evaluating the flow dynamics and viability indices of cancer cells in a constricted microchannel. For this purpose, metastatic cervical cancer (HeLa) cells were used as a model system to examine metastatic flow, where the cells with larger dimensions were seen to deform and traverse through microcapillaries. CTCs of HeLa have been studied by several other researchers recently^3,34,35^. To emulate the conditions, a microchannel having both converging and diverging sections with a constricted portion was fabricated using polydimethylsiloxane (PDMS), and the open surface was sealed with a glass cover-slide. HeLa cells were allowed to pass through the constricted channels of width 7 μm for a distance of 200 μm at a constant peristaltic flow rate (30 μl/h). Physical parameters such as entry time, transit velocity and elongation index of a single HeLa cell were evaluated using representative images deconvoluted from high-speed videographs. Biological parameters, such as the viability of cancer cells, were confirmed by the dye exclusion process and, most importantly, persistence of metastatic onset of HeLa cells to retain virulence behaviour was also observed by matrix metalloproteinase-2 gene expression analysis. To the best of our knowledge, this is the first experimental report to demonstrate the existence of live and metastatic populations of cancer cells after passing through a constriction, which could have a significant impact on revealing the therapeutic modalities of metastatic cancers in the near future. The crux of the current concept is illustrated in the Fig. 1.

## Results

### Characterisation and flow profile of HeLa cells through a constricted microchannel

**Figure 2:**
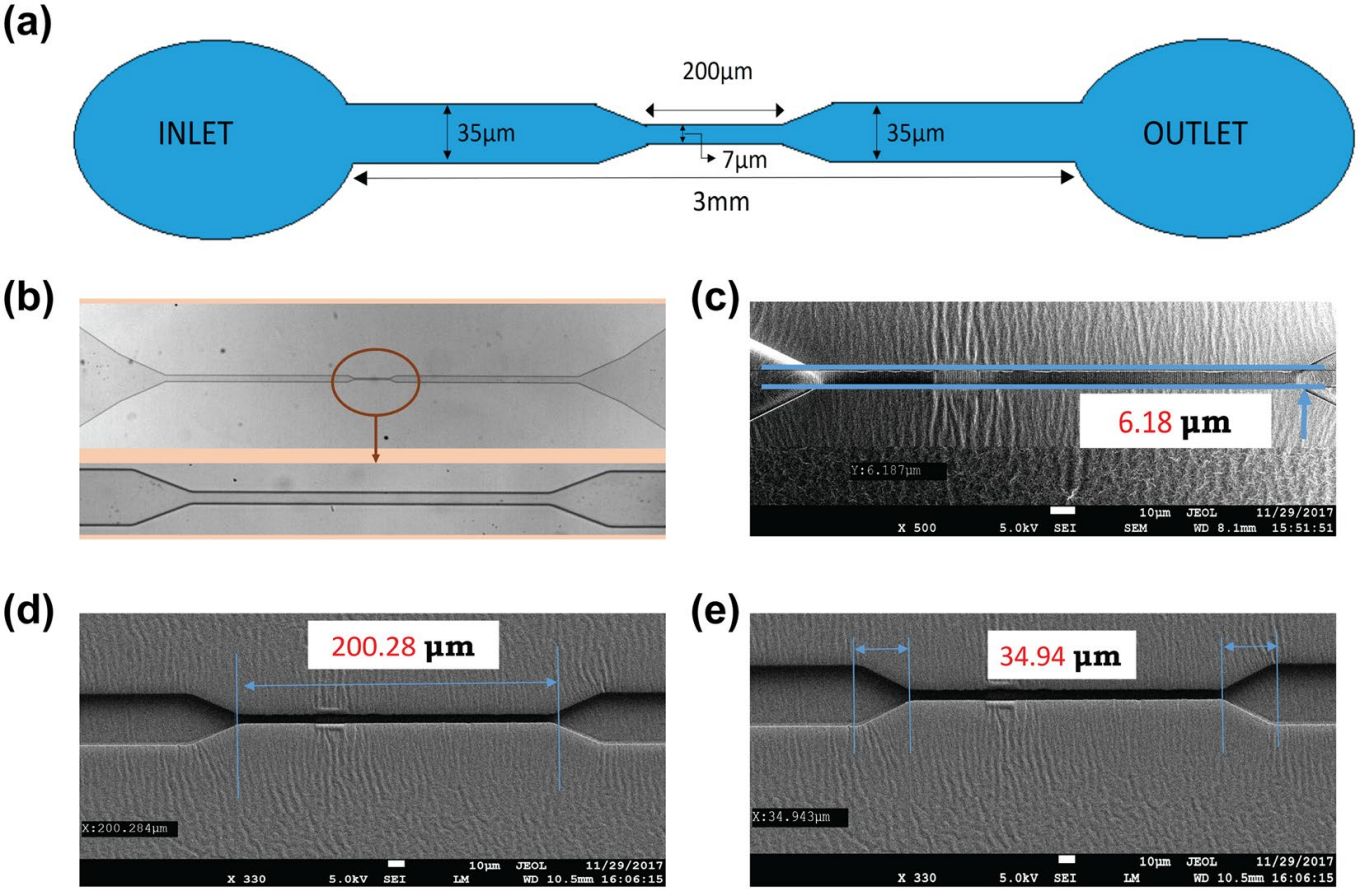
Design and characterization of the microchannel. (**a**) Design of the microchannel; (**b**) Microscopic view of the microchannel with a magnified view of the constricted section; (**c**) FESEM analysis of the width of the constricted passage; (**d**) FESEM analysis of the length of the constricted passage; (**e**) FESEM analysis of the length of the tapered section on either side of the constricted section.

The design of the microchannel is shown in Fig. 2a. The architecture of the fabricated PDMS-based microchannel was visualized under a bright-field microscope (Fig. 2b). The lower panel in Fig. 2b shows a magnified view of the constricted portion of the channel. The precise width and length of the constricted section were ascertained by Field emission scanning electron microscopy (FESEM) analysis and were found to be 6.18 and 200.28 µm, respectively, as shown in Fig. 2c and d. The tapered sections were marked to show the entrance and exit portions of the constricted passage (Fig. 2e), with a total length of 34.94 µm. The width of the channel was considered to be 7 µm for the entire study.

**Figure 3:**
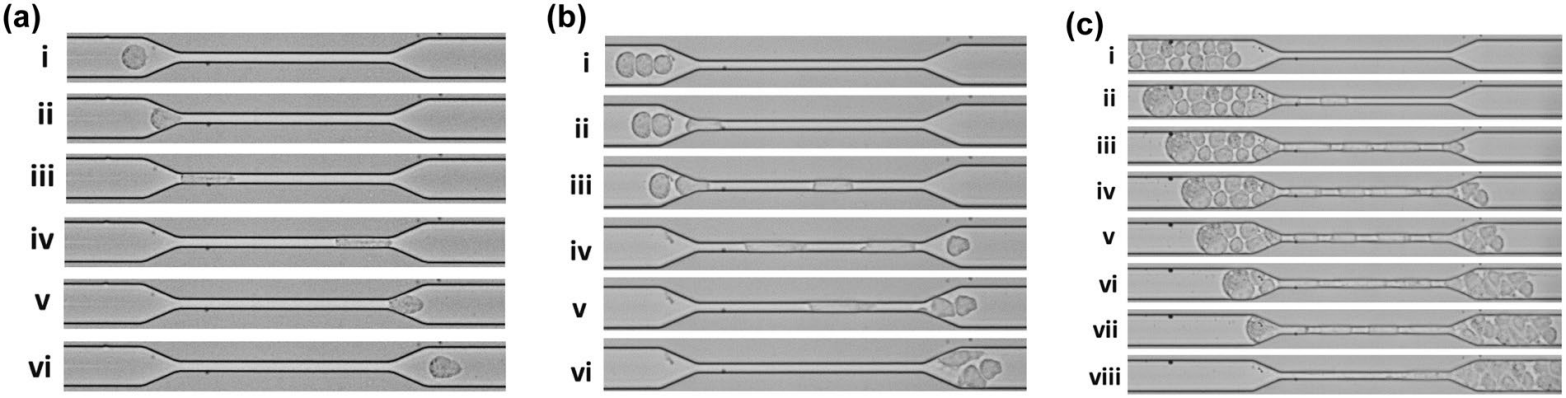
High-speed camera imaging of the movement of HeLa cells through the constricted portion of the microchannel. (**a**) Motion of a single cell: (i) a single drop approaching the constriction, (ii) cell entering the constriction, (iii) and (iv) cell moving inside the constriction, (v) cell squeezing out of the constriction and (vi) cell flowing outside the constriction. (**b**) Motion of three cells: (i) three cells approaching the constriction, (ii) and (iii) cells entering the constriction one by one, (iv) and (v) cells travelling inside the constriction and moving out one by one and (vi) cells flowing outside the constriction. (**c**) Motion of aggregated cells: (i) aggregated cells approaching the constriction, (ii) and (iii) cells entering the constriction in a queue and travelling through it and (iv–viii) cells entering through the constriction and moving out one by one to re-form the aggregated cluster of HeLa cells.

The experimental setup was calibrated with a continuous flow of PBS for 30 min. Then 1 × 10^5^ HeLa cells/ml were allowed to flow through the microchannel at an optimum flow rate of 30 µl/h. The cells moved through the constricted passage of the microchannel at high speed. The motion of the cells was video-graphed by Phantom MIRO-LAB320 High-speed camera at 50000–60000 fps using 20X objective in Leica DMI3000 M Microscope. The video-graph provided in the Supplementary Video S1 had captured the entire channel from the inlet to the outlet reservoir. It was observed that the HeLa cells appeared as singlets as well as loosely bound aggregates in the inlet reservoir. Figure 3a–c illustrate that HeLa cells of approximate size 20 µm were deformed and elongated extensively while passing through the constriction of width 7 µm. Figure 3a(i–vi) and Supplementary Video S2 show time-lapse movement of a single HeLa cell through the constricted passage. Figure 3a(i,ii) show that the cells were squeezed slowly and then entered the constriction. Similarly, enhanced deformation and elongation of the cells were observed in Fig. 3a(iii,iv) while moving inside the constriction. Once the cells had moved out of the constriction, they gradually returned back to shape, as shown in Fig. 3a(v). Finally, the cells moved further downstream of the channel and almost regained the original shape, as shown in Fig. 3a(vi). Movement of three HeLa cells through the constricted portion of the channel is shown in Fig. 3b(i–vi) and Supplementary Video S3, where the cells entered one at a time and passed through the constriction in a moving queue. The movement of aggregated HeLa cells through the constricted channel was also recorded and is shown in Fig. 3c(i–viii) and Supplementary Video S4. It was observed that many cells were aggregated and connected with each other. However, the cells underwent continuous reorientation and rearrangement while entering the constriction and in this continuous process, the cells got detached from each other. As a result, many cells appeared to be disconnected or loosely bound in the entry region, whereas they aggregated again after leaving the constricted portion.

### Estimation of flow parameters of a single HeLa cell

**Figure 4:**
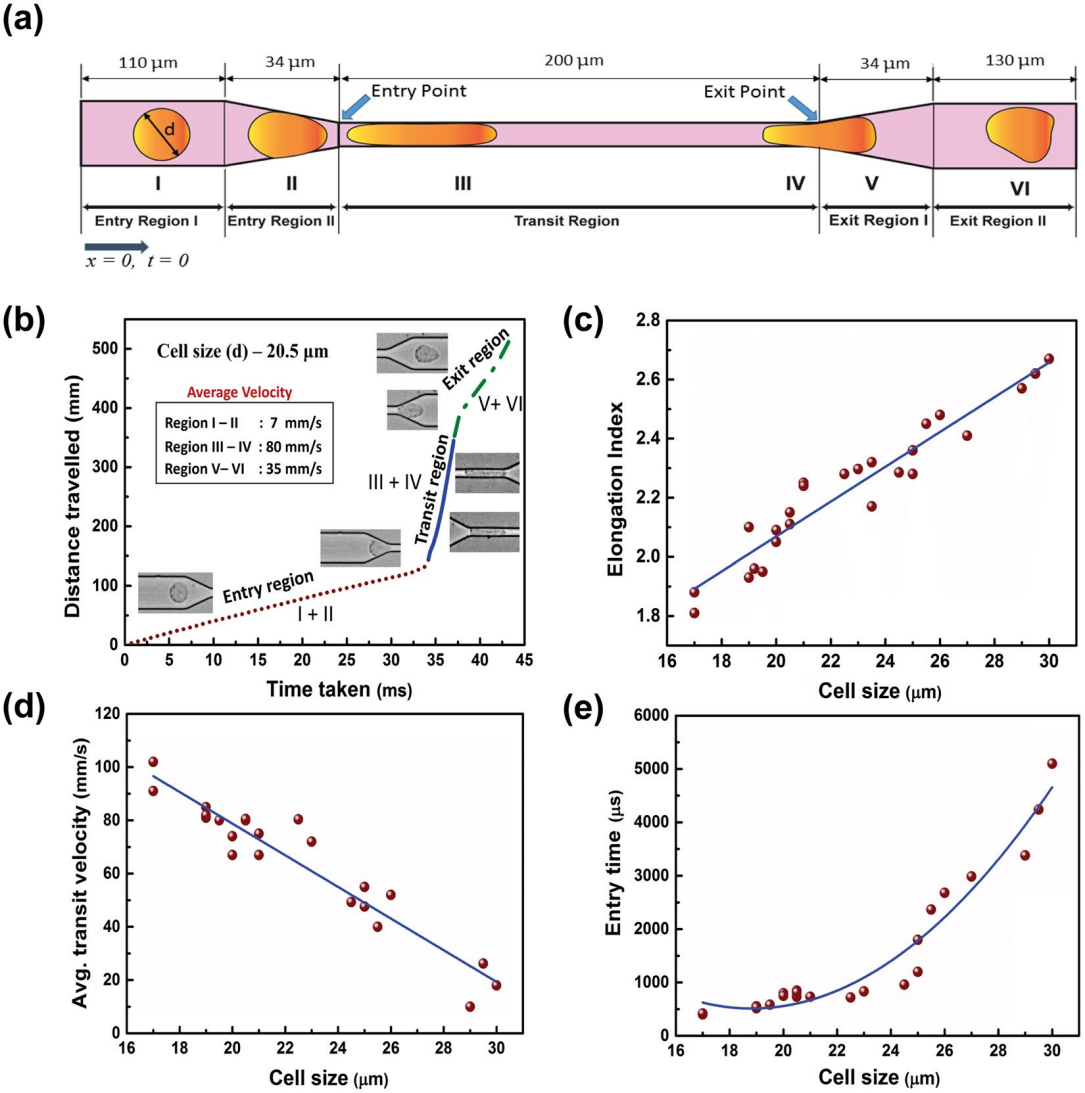
Movement of single cells in the microchannel. (**a**) Schematic representation of the motion of a single cell through different sections of the constricted channel. (**b**) Motion of a single cell of diameter 20.5 µm. (**c**–**e**) Elongation index, transit velocity and entry time, respectively, of single cells with diameters ranging from 17 to 30 µm.

The dynamics of motion and deformation of HeLa cells through a constricted channel were analysed from the movement of single cells of different sizes (17–30 µm). Still images were obtained from the recorded videos at the required time frames. “Image J” software was used for image processing, which enabled us to measure the diameter and length of the cells. The scale for measurement was set from the known dimensions of the channel. Physical parameters, such as entry time, transit velocity and elongation index, of a single HeLa cell were calculated based on the positioning of a cell in different sections of the microchannel, as portrayed in Fig. 4a. The domain of interest was divided into three sections: Entry, Transit and Exit regions. The Entry and Exit regions were subdivided into Entry regions I and II and Exit regions I and II, respectively. At the beginning of Entry region I, the time (t) and distance (x) travelled by a cell were taken as *t* = 0 and *x* = 0. Entry region I was 110 μm long and 35 μm wide, where cells were found to retain their original shapes. Subsequently, the cells entered the converging section of length 34 μm denoted Entry region II. The cells were mostly deformed in this region and prepared to enter the constriction ahead. Next, the cells moved to the Transit region, with a length of 200 μm and a width of 7 μm. The cell deformed substantially in this region, undergoing extensive elongation. They then moved out to the Exit region I of length 34 μm, which was a diverging section where the cells tried to recover their shape. In Exit region II, with a length of 130 μm and a width 35 μm, the cells continued to regain a circular shape while traversing downstream. The different positions of the cell are marked I, II, III, IV, V and VI, as shown in Fig. 4a.

Figure 4b shows the dynamics of a single cell of diameter 20.5 μm traversing through the different regions. The slope obtained from the distance travelled versus time graph revealed that the cell moved very slowly in the Entry region, where it approached the constriction and squeezed itself to enter it. The slope was very steep in the transit region, where the cell became elongated and moved with a high velocity. In the Exit region, the cells again slowed down while trying to retrieve their shape. The average cell velocity was calculated to be 4, 70 and 26 mm/s in the Entry, Transit and Exit regions, respectively.

The deformation, velocity and entry time of the cells were calculated considering the diameter of cells ranging from 17 to 30 μm. Figure 4c–e show the scatter plots for the different parameters using cells of varied sizes. The best fitted curves for the data points in the graphs are depicted by the blue lines. As the cells became elongated and deformed on entering the constricted channel, the elongation index of the cells was measured with respect to the original diameter and is given by-

$${\rm{Elongation}}\,{\rm{index}}=l/d$$

where, *l* = maximum length of the cell in the microchannel and *d* = original diameter of the cell.

Figure 4c shows that the deformation and elongation of large cells were greater than those of small cells while transiting through the constriction. It was observed in Fig. 4d, based on the calculation of the transit velocity that a small cell passed through the constricted passage much faster than a large cell. The time taken by the HeLa cells to enter the constriction is shown in Fig. 4e. It was noted that large cells took longer time than small cells to squeeze and accommodate themselves in the constricted passage.

### Assessment of cell viability

**Figure 5:**
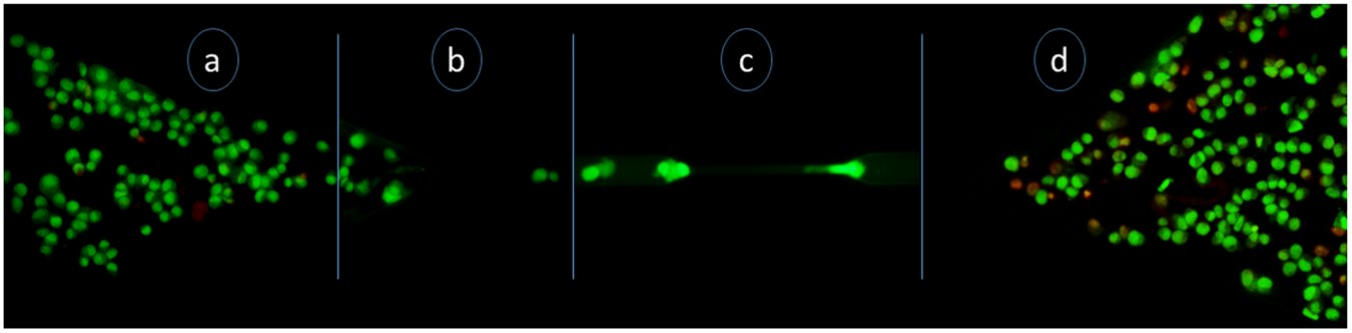
Representative images of AO/EtBr dual staining in different sections of the channel. (**a**) Live cells in the inlet section (with some debris); (**b**) live cells entering the channel from the inlet; (**c**) a live cell moving in the constricted portion of the channel; and (**d**) live and dead cells in the outlet section.

It was important to explore the survival ability and mortality rate of the cells as they passed through the 7 µm constricted portion of the microchannel to address stimulated metastatic flow. Representative images after AO/EtBr dual staining are shown in Fig. 5, for cells in different sections of the constricted microchannel. It is evident from Fig. 5a that the cells were live and stained green, with hardly any dead cell (visible in red), in the Inlet section of the channel. Figure 5b shows live cells entering the channel from the inlet, and Fig. 5c shows a green deformed live cell that had just entered the constricted portion. From Fig. 5d, it is observed that, although the number of dead cells increased in the Outlet section, a significant population of live cells (around 50%) also existed. From this experiment, it was inferred that a portion of HeLa cells died during extensive deformation and elongation, but a moderate fraction of HeLa cells still retrieved their original shape and remained alive even after passing through the constriction. Such a population of live cells could represent the survival of metastatic cancer cells in harsh circulating conditions through microcapillaries in the human body. The accumulation of a high population of dead cells (stained red) at the outlet was possibly due to extensive deformation of cells leading to membrane compromisation while passing through the constricted channel.

### Evaluation of viability (HeLa cells) by the Trypan Blue method

Live or dead cells were also analysed using the Trypan Blue dye exclusion assay: live and healthy cells were unstained or excluded from dye, whereas dead or membrane-compromised cells appeared blue due to Trypan Blue retention. The stained cells were analysed in the cell counting device and the number of live and dead cells were calculated using the Countess automated cell counter. To study the viability of the cells passing through constriction, adequate number of cells were collected from the outlet of the channel, which consisted of singlets as well as aggregates.

**Figure 6:**
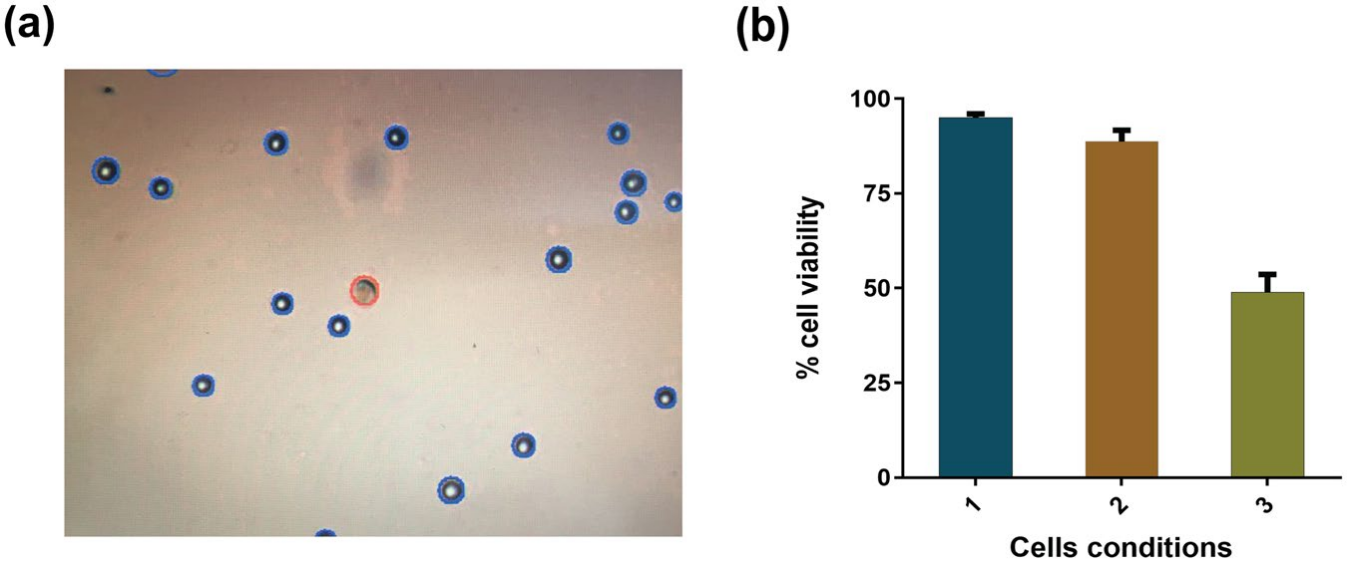
Viability of HeLa cells passing through the constricted channel. (**a**) Image of stained cells obtained from the Countess automated cell counter. (**b**) Comparison of percentage of cells still viable after passing through the constriction with the initial conditions and with cells maintained in the same environment for same period of time but not passed through the constriction. Cell condition 1 refers to the initial cells, cell condition 2 refers to mock experimental control cells without passing through the channel and cell condition 3 refers to the cells collected from the outlet of the constricted channel.

Figure 6a illustrates the stained cells obtained from the Countess automated cell counter, showing live and dead cells. The graphical representation of the results is presented in Fig. 6b. The results shown in Fig. 6b for viability index were obtained from three independent set of experiments. It was observed from the Trypan Blue dye exclusion assay that the initial conditions of the HeLa cells (before entering the constricted channel) were such that 95% of the cells were alive. When the cells suspended in the medium were allowed to pass through the constricted channel, around 50% of the cells retained their properties well and were alive. This corroborates the previous fluorescence-based study and gives a quantitative idea about the viability of the cells.

### Metastatic profile

**Figure 7:**
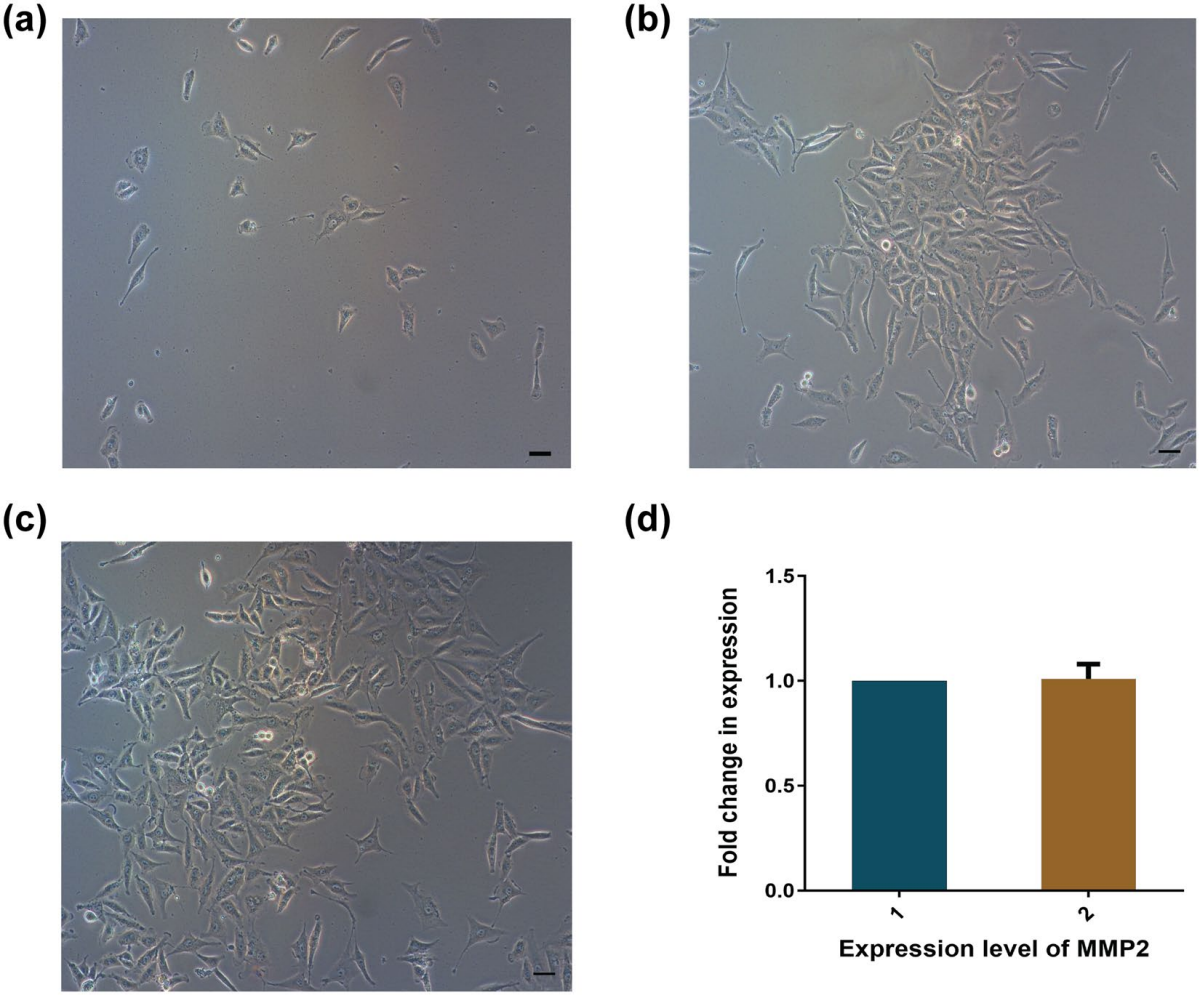
Metastatic profile of HeLa cells. Microscopic images of the cells collected from the outlet of the constricted channel and regrown in a cell culture dish for (**a**) 12, (**b**) 48 and (**c**) 72 h; and (**d**) representation of expression level of MMP2. Bar = 50 μm.

The experimental evidences confirmed the ability of HeLa cells to change morphology by deformation for crossing a 7 µm constriction and remained mostly alive. Further, HeLa cells that accumulated at the outlet were collected and allowed to adhere to a culture disc to access the metastatic profile. Figure 7a depicts the adherence of cells, collected from the outlet, by 12 h. The cells started to divide and formed colonies (Fig. 7b) by 48 h, and became confluent at 72 h (Fig. 7c). The sequence of images (Fig. 7a–c) indicates the capability of HeLa to regrow and to form colonies even after passing through a harsh constricted passage.

In the second line of investigation, the expression of the protein MMP2, involved in the degradation of the matrix enabling cells to be released into the bloodstream^36^, was evaluated. In HeLa cells, the expression of MMP2 protein is considerably higher than in non-cancerous cells^37^. Hence qPCR was performed on the cDNA obtained from the total RNA of HeLa cells grown for 72 h after collection from the inlet and outlet of the setup. The qPCR (Fig. 7d) showed that the expression of MMP2 under both conditions remained unaltered. The results shown in Fig. 7d were accumulated from three independent set of experiments. Such fascinating results confirmed that the cells hardly lost any virulence and retained their capability to adhere, forming colonies that are reminiscent of CTC-like behaviour.

## Discussions

In this experimental work, flow dynamics and survival ability of cancer cells transiting through microcapillaries that mimic the microvascular environment has been elucidated. In contradiction to the decades-held assumption that owing to the difference in size between the CTC diameter and diameter of capillaries, the clusters are incapable of traversing through microcapillaries^38^, we found here that the Hela cells are capable of migrating through microcapillaries by undergoing immense deformation. Although, a similar investigation on motion of CTCs by Au *et al*.^31^ showed deformation of CTCs traversing through capillaries, but there was no experimental evidence on either cell viability or metastatic gene expression profile to explain biomimetic conditions. To the best of our knowledge, this paper provides the first evidence that majority of the cancer cells retain sufficient viability and are potent enough for causing metastasis at distant sites even after undergoing drastic changes in the morphology due to constriction.

PDMS microchannel of minimum constricted width of 7 μm for a distance of 200 μm in a total length of 3 mm was fabricated that possessed close analogy in terms of dimensions with human capillaries (width in the range of 5–10 μm). The laminar flow behaviour as well as the elastic properties of the PDMS walls are similar to that of the blood vessels and capillary walls, and both stand very important while studying rheological deformation of cells^39^. The FESEM characterisation of the channel ensured that the fabricated channel actually possessed the dimensions as per our design.

The remarkable ability of singlets and even aggregated cluster of HeLa cells to transit through capillary sized micro-constriction was witnessed by capturing videos through high speed camera. It was observed that the key to aggregated HeLa cells transit through narrow capillaries could be due to their ability to rapidly unfold into single file chains while entering into the constriction, which significantly reduces their overall resistance to flow. The associated HeLa cells segregated as individual cells in tandem while traversing through the microcapillary and reorganized themselves to aggregate again after exit. The high speed videographs were deconvoluted to obtain still images and crucial parameters like cell elongation index, average transit velocity and entry time of the single cells passing through constriction were calculated, to understand the dynamics of HeLa cell motility. This detailed investigation of the hydrodynamic parameters of HeLa cells of varied sizes helped us in finding a common trend in which the cells deform and move inside the constriction with respect to their sizes. The values of the different hydrodynamic parameters obtained in this study correspond to the optimized flow rate of 30 μl/hr for our experiments. The extensive change in morphology of the cells witnessed while transiting through constriction raised our inquisitiveness to find out whether the cells that exit through the constriction could retain their metastatic potency. This query motivated us to extend our study in the direction of addressing some crucial biological behaviour of these transiting HeLa cells that would help us to understand the potency of cancer cells to metastasize at distant sites passing through microcapillaries.

Reasonable viability of the cells after passage through the constriction, determined by dual AO/EtBr staining, was substantiated by Trypan Blue dye exclusion assay that confirmed the retention of around 50% viable HeLa cell population at the outlet. Data from the mentioned experiments suggested that the HeLa cells, even after passing through the constriction, remained viable to form further tumours at extremities of the body. In other set of experiments, we successfully collected the cells from the outlet of the constricted channel and grew the cells in cell culture. In 48 h of cell culture, the cells started to divide and formed colonies and by the end of the 72 h they achieved confluency. This experiment assured that, although most of the cells died during extensive deformation and elongation, yet a substantial population of live cells was found to exist, which retained the capability of portraying the phenomenon of metastasis further. Moreover, Real-time measurement of MMP2 expression to determine important metastatic biomarker of HeLa cells opened up a new paradigm to understand the possible dynamics of cancer cells to spread metastasis by traversing through microcapillaries in human body. This is the first instance where our experimental findings on HeLa cell motion might enable to develop futuristic protocols for cancer theranostics.

## Materials and Methods

### Reagents and chemicals

All molecular grade chemicals and reagents were purchased from Sigma-Aldrich, unless mentioned otherwise.

### Cultivation of HeLa cells

HeLa cervical cancer cells obtained from the repository of the National Centre for Cell Science (NCCS), Pune, India, were cultured in Dulbecco’s modified Eagle’s medium (DMEM high glucose), containing 10% fetal bovine serum (FBS) and 1% penicillin/streptomycin (100 U/ml; 0.1 mg/ml), in humidified 5% CO_2_ at 37 °C. For the purpose of our experiments, confluent HeLa cells grown on a 60 mm cell culture dish were washed thoroughly with phosphate-buffered saline (PBS) and trypsinized with 80 µl of trypsin–EDTA for 2–3 min. After detachment, the cells were suspended in 1 ml of DMEM and examined under a microscope to ascertain intactness. Finally, cells taken in a 1.5 ml Eppendorf tube were used for further procedures.

### Fabrication of microchannels

According to the design shown in Fig. 2a, a Su8 master silicon wafer was prepared in the CeNSE Department of IISc Bangalore, India, having imprints for 16 channels in the single master. The open channels were made of PDMS. To prepare PDMS solution, SYLGARD 184 silicone elastomer was mixed with a cross-linker in a ratio of 10:1. To make the mould, a nylon ring was placed carefully over the master placed on top of aluminium foil and the PDMS solution was slowly poured over the master bounded by the nylon ring. The mould was then placed over aluminium foil and held over a hot-plate for 30 min. Upon solidification of PDMS on the mould, the entire array was kept at room temperature for cooling. The PDMS structure with several integrated channels was then peeled off and, finally, individual channels were formed after surgical sectioning. The inlet and outlet of each channel were developed by making appropriate holes through punching. The top portions of the open channels were then sealed using glass slides by treating them inside an oxygen plasma chamber for 1 minute in a clean environment. The PDMS open channels were then fixed with the glass slides to form closed channels.

### Experimental setup

The microchannel was placed on the stage of the microscope and semi-rigid Polyethylene tubing of inner diameter 0.38 mm and outer diameter 1.09 mm (Prolab Marketing, India) was connected to the inlet and outlet. The concentration of cells used in the experiments was around 1 × 10^5^ cells per ml of medium, in which approximately 95% of the cancer cells were live initially in all the experiments. The cells were transferred from the Eppendorf tube to a syringe, which was fixed securely in the syringe pump. The connections to the inlet and outlet of the channel were secured well with close observation. When the entire setup was completed, the microscope and pump were connected to a power source. As the syringe pump started to operate, the cells suspended in DMEM medium started to fill the connecting tube and flow through the microchannel. The average velocity of blood (usually measured in cm/s) generally varies from 0.03 to 40 cm/s as the blood flows through the vena cava, capillaries and aorta^40^. The cells suspended in media were allowed to flow passively at constant flow rate of 30 μl/hr. As per the given flow rate, the average velocity of the external fluid in the constriction (7 µm width) was found to be 17 cm/s and in the rest of the channel (width 35 µm) the velocity was 3.4 cm/s. The flow gradually became steady and the motion of the cells were observed and recorded at a high frame rate of 50000–60000 fps using the video module of the Leica LAS software. The videos were then deconvoluted to obtain images at required time instants.

### Dual staining

Morphological identification of live, apoptotic and necrotic cells was performed by dual staining with Acridine Orange (AO)/ethidium bromide (EtBr). EtBr stains only the DNA of the dead or membrane-compromised cells whereas AO stains all the cells. HeLa cells were taken in a 1.5 ml tube and stained with a mixture of 2 µg/ml AO and 6 µg/ml EtBr and kept in the dark at 37 °C. After 10 min, the cells were centrifuged at 650 rpm for 5 min and the pelleted cells were redispersed in the DMEM medium for further processing. The cells were visualized under an epi-fluorescence microscope (Eclipse Ti-U, Nikon, Tokyo, Japan) using 10X objective. Excitation filter of 480/15 nm was used to collect green fluorescence of AO, whereas 540/25 nm excitation filter was used for collecting red fluorescence of EtBr.

### Trypan Blue staining

Trypan Blue dye is used to stain membrane-compromised or dead cells, whereas live cells exclude the dye. HeLa cells were seeded at a density of 1 × 10^5^ in a 60 mm cell culture dish in the presence of DMEM medium. Confluent cells were washed with PBS and resuspended in fresh DMEM medium in an Eppendorf tube. Equal volumes of Trypan Blue dye (10 µl) and the cells were mixed and loaded on the counting chamber. The viable cells (%) were counted using a Countess automated cell counter (Invitrogen). The images highlighting the live and dead cells were also captured using the same instrument.

### Virulence study

A virulence study was performed to investigate whether the HeLa cells retain their metastatic ability after passing through the channel. For this, HeLa cells were grown in a 60 mm cell culture dish and subsequently harvested. The cells were collected in an Eppendorf tube after passing through the channel and tube. The collected cells were dispersed in DMEM medium in a 60 mm cell culture dish, regrown and observed at 10X magnification under a bright field microscope (Eclipse Ti-U, Nikon, Tokyo, Japan) for 12, 48 and 72 h.

### Metastatic gene expression analysis

Matrix metalloproteinases (MMPs) are a group of enzymes that are mostly responsible for oncogenesis, growth and normal tissue turnover by degrading most of the extracellular matrix^36^. MMP 2 expression was examined using quantitative real-time PCR (qPCR) in HeLa cells before and after passing through the microchannel. After passing through the microchannel, the cells were collected and grown to confluency on a culture plate. Further, the cells were lysed and total RNA was isolated. Total RNA (1 µg) was used to prepare cDNA using a Verso cDNA Kit (Thermo Scientific). qPCR was performed using the MMP2 primers and SYBR Green as reporter dye (Power SYBR Green PCR master mix, Applied Biosystems) in a Rotor-Gene Q (Qiagen). The relative MMP2 mRNA expression was calculated by the ΔΔCt method using GAPDH, the endogenous control.

## Electronic supplementary material

Supplementary video S1

Supplementary video S2

Supplementary video S3

Supplementary video S4

Supplementary Information
