# Supplementary material for: Understanding flow dynamics, viability and metastatic potency of cervical cancer (HeLa) cells through constricted microchannel
Source: Sci Rep. 2018 Nov 26;8:17357. doi: 10.1038/s41598-018-35646-3 (PMC6255798; doi:10.1038/s41598-018-35646-3)
Supplement: Supplementary file 5 — Supplementary Information [file 41598_2018_35646_MOESM5_ESM.pdf]

Supplementary Information

Understanding flow dynamics, viability and metastatic potency of cervical cancer (HeLa) cells through constricted microchannel

Binita Nath[a], Asif Raza[b], Vishal Sethi[a], Amaresh Dalal[a], Siddhartha Sankar Ghosh[b] and Gautam Biswas[a*]

[a] Department of Mechanical Engineering, Indian Institute of Technology, Guwahati – 781039, India

[b] Department of Bioscience and Bioengineering, Indian Institute of Technology, Guwahati - 781039, India

Correspondence should be addressed to: Gautam Biswas, Department of Mechanical Engineering, Indian Institute of Technology Guwahati, Guwahati-39, Assam, India

Ph no.: +0361-258-2001; Fax: +0361-269-2321; Email: gtm@iitg.ernet.in

Supplementary video S1 shows the entire channel from the inlet to the outlet reservoir.

Supplementary video S2 shows the motion of a single HeLa cell through the constricted channel.

Supplementary video S3 shows the motion of three HeLa cells through the constricted channel.

Supplementary video S4 shows the motion of aggregated HeLa cells through the constricted channel.
